# A rapid clinic-based service for an emergency department of a tertiary teaching hospital during a dengue outbreak

**DOI:** 10.1097/MD.0000000000025311

**Published:** 2021-04-09

**Authors:** Hsin-I. Shih, Yi-Ting Huang, Chih-Chia Hsieh, Tzu-Ching Sung

**Affiliations:** a Department of Emergency Medicine, National Cheng Kung University Hospital; b School of Medicine; c Department of Public Health, College of Medicine, National Cheng Kung University, Tainan; d School of Medicine for International Students, College of Medicine, I-Shou University, Kaohsiung, Taiwan.

**Keywords:** dengue, emergency department, outbreak, Taiwan

## Abstract

The 2015 dengue outbreak in southern Taiwan turned into a public health emergency, resulting in a large-scale mobilization of personnel from the emergency department (ED) services operating in and near full capacity to assist with the outbreak. This study aimed to assess a rapid independent clinic-based service (RCS), which was set up and designed to relieve the overcrowding of the regular ambulatory and emergency services during an epidemic of dengue.

This is a retrospective cross-sectional study.

National Cheng Kung University Hospital, Tainan, Taiwan.

Patients with positive test results were enrolled and reviewed to evaluate the efficacy of RCS implementation between August and October 2015. The case-treatment rates stratified by length of stay (LOS) were used to examine the performance of the RCS that was set up outside the ED and designed to relieve the overcrowding of the regular ambulatory and emergency services.

Patients with dengue-like illnesses may arrive at the hospital and require optimal ED triage and management thereafter. Although the outbreak resulted in a shortage of spare space in the ED, a proper response from the hospital administration would ameliorate the work overload of the staff and would not decrease the quality of care for critical patients.

An early and restrictive intensive intervention was beneficial to health care facilities during a dengue outbreak. Further planning and training of the RCS could be crucial for hospital preparedness for infectious disease outbreaks.

## 1 Introduction

The global emergence of dengue virus (DENV) in the recent decades has caused the substantial health and economic burdens on patients and healthcare systems, especially in the tropical and subtropical regions. Each year, >250,000 cases of dengue hemorrhagic fever and dengue shock syndrome (DSS) are reported from an estimated 50 million dengue infections.^[1–3]^ Dengue is transmitted between people by the mosquitoes *Aedes aegypti* and *Aedes albopictus* throughout the world. Severe dengue-related fatal cases typically present with DSS, and the mortality rate for DSS is reportedly 50 times higher than that in dengue patients without DSS.^[1,4]^ The reported annual dengue epidemic in Taiwan, located in the tropical and subtropical areas of the Western Pacific Rim, included imported and domestic cases ranging from 3000 to 10,000 cases per year. Several large-scale dengue outbreaks with more than 10,000 cases occurred in the past two decades in one of the major cities, Kaohsiung, with a substantial number of severe dengue type 2 cases in 2002 and dengue type 1 in 2014.^[5]^

In 2015, there has been a recent large-scale dengue outbreak severely affected another major city in southern Taiwan, Tainan, which is a city with a population of 1.8 million and with 780,000 people living in the metropolitan area. Historically, the number of annual dengue cases in Tainan was approximately 1,000. During this large outbreak, approximately 43,000 patients and 130 fatal patients were observed from August 2015 to February in 2016.^[6]^ The large census of patients rapidly flooded hospitals that led to the many emergency department (ED) services operating in and near full capacity. Additionally, a crowded ED resulted in a heavy workload for the health care professionals.

The average length of stay (LOS) per patient measured from the patient's arrival to leave has been shown to be a surrogate indicator of crowding in the ED. It is frequently considered a key process indicator for performance improvement and clinical and operational efficiency.^[7]^ The association between LOS and throughput and output factors related to the conceptual model of ED crowding has been proven. The increased number of additional ED admissions for all shifts was associated with an increase in LOS.^[8]^ The LOS, characteristic of the retrospective nature, was used to measure Taiwan's hospital clinical performance indicators.^[9]^

More recently, a rapid flu clinic service has been applied in this hospital to decrease the risk of cross-transmission of infections and the negative impacts of the overcrowded emergency department in 2009.^[10]^ The rapid clinic service could be extended in other circumstances and still needs further testing. Therefore, adapting a similar concept to propose an independent unit to initiate a rapid clinic-based service (RCS) that can be used to improve the triage process is critical to the health care facility and health care system at large. This study aimed to investigate the impact of an independent RCS that was set up outside the ED and designed to relieve the overcrowding of the regular ambulatory and emergency services as well as a sharply increased hospital census in response to the ED services during the overcrowding periods.

## 2 Methods

### 2.1 Ethics and patient selection

Patients and the public were not directly involved in the design, conduct, or reporting on this study.

All provisions of the study were in accordance with the Helsinki Declaration. To protect personal privacy, the electronic databases were decoded for research purposes. Patient information was collected and made anonymous and patients were deidentified before analysis; therefore, the requirement for informed consent was waived by the institutional review board (IRB). Both of the study protocols and data have been approved by the IRB of National Cheng Kung University Hospital (A-ER-104-223).

### 2.2 Study design and setting

We conducted a retrospective cross-sectional study from August 2015 to December 2015 in a tertiary teaching hospital in southern Taiwan outfitted with 1000 beds, 100 of which were intensive care unit beds. The modified Canadian Triage & Acuity Scale was applied as the triage tool (Taiwan triage and acuity scale).^[11]^ The annual number of patients treated in the study ED was approximately 89,700 in 2014. The mean LOS in the ED and the LOS for patients who left following treatment in the ED were 6.89 hours and 4.8 hours, respectively, in 2014. To shorten the unnecessary LOS for mild dengue patients, we established an independent RCS in a tertiary hospital to provide rapid dengue tests and counseling beginning in August 2015.

**Figure 1:**
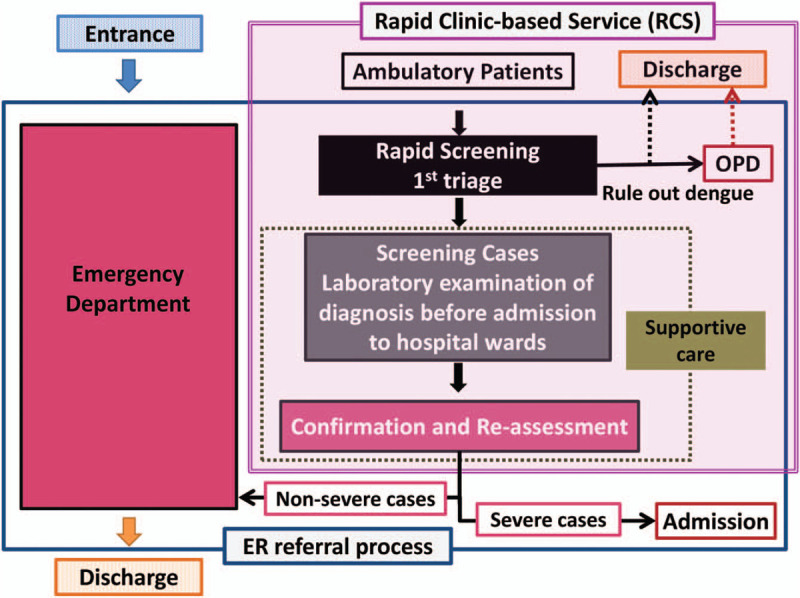
Design of the management for RCS during the 2015 Dengue outbreak. RCS = rapid clinic-based service.

RCS has been built since 2012 and was designed to treat large numbers of ambulatory or special hazard exposure (Nuclear, Radiation, Biology, and Chemical, NRBC) patients. Ambulatory patients must be present at the RCS and have their history taken and undergo a physical examination, including rapid dengue diagnosis, that is, NS1, IgM/IgG tests, and receive essential treatment, including a complete examination of the patient's blood and blood supply. Figure 1 depicts the flow chart of an independent unit (RCS) within the ED set up from August to October 2015. All patients initially admitted to the hospital with dengue-like illnesses or symptoms went directly to RCS and centralized in the RCS instead of triaged at the ED. Patients ruled out for rapid screening after first triage were transferred to an outpatient clinic or directly discharged from RCS. After complete evaluation of laboratory tests and further assessment, patients who had dengue but not severe will be returned to ED, and who were confirmed to have severe dengue will be admitted directly from RCS.

The RCS regularly operated 16 hours per day and was staffed by 1 physician and 2 nurses. Additionally, the RCS was staffed by rotating physicians from all clinical departments during day and evening shifts (9:00–18:00). During night shifts (18:00–21:00), emergency physicians (EPs) took over the work. Additional physicians who joined the RCS were from the departments of internal medicine, family medicine, and neurology to alleviate ED overcrowding and offload clinical duties onto EPs so that they could focus on the care and management of critical emergency patients. Each non-EP took a 3-hour shift (9:00–12:00; 12:00–15:00; 15:00–18:00; 18:00–21:00) and evaluated patients who had mild dengue-like illnesses or symptoms, that is, fever, generalized bone pain, and muscle soreness. A predesigned computerized medical record, including basic demographic information, underlying diseases, important physical examinations, a history of stay, and recent travel history and prescription packages, such as the diagnosis package and treatment formula, were set up for physicians and nurses to rapidly access and treat these patients.

### 2.3 Dengue diagnosis and treatment

Five to 10 mL blood samples were drawn to test complete blood counts, renal and liver functions, and dengue virus concentrations in the blood of the subjects. About 5 to 10 μL of serum was drawn to detect nonstructural protein 1 (NS1) and the IgM/IgG tested by using the SD BIOLINE dengue DUO rapid immunochromatographic test kit (Standard Diagnostics, Inc., Kyonggi-do, Korea).^[12,13]^ A real-time reverse transcription polymerase chain reaction (rRT-PCR) test was performed using the same method as previously described. In addition to NS1, IgM/IgG or RT-PCR tests designed to diagnose dengue, other examinations such as cultures, chest x-rays or image studies were available based on the clinical judgement of the physicians.

### 2.4 Statistical analysis

The ED performance was evaluated by LOS and the case-treatment rates with respect to 4 categories of the LOS, that is, ≤ 2 hours, 2 < LOS ≤ 6 hours, 6 < LOS ≤ 24 hours, and > 24 hours, respectively. The duration of the recruitment of ED admissions for the case-treatment was calculated from the patients who arrived at the hospital who were registered and triaged to the first assessment and necessary treatment done by the health care workers. Case-treatment rates, defined as the registered and triaged ED patient visits with respect to each of the four LOS categories divided by the total number of registered and triaged ED patient visits, were estimated to assess the impact of RCS on ED performance. Furthermore, case-treatment rates in the four LOS categories, that is, ≤2 hours, 2 < LOS ≤ 6 hours, 6 < LOS ≤ 24 hours, and >24 hours, were compared for dengue (August to October in 2014 and 2016), dengue-outbreak (August to October in 2015), and non-dengue seasons to evaluate the effectiveness of dengue rapid clinical services.

Descriptive statistics and an independent 2-sample *t* test were calculated for LOS of triaged patients for three major divisions of a tertiary hospital. An independent 2-sample *t* test compares the mean LOS during the dengue-outbreak and non-dengue seasons in 2015 to determine whether there is a statistical difference between the two groups. Logistic regression methods were used to analyze the effect estimates of case-treatment rates for 4 LOS categories during the January 2014 and September 2016 seasons, which helped with forecasting, modeling, and calculating odds ratios of dengue and dengue-outbreak seasons in comparison to non-dengue seasons. The lower and upper 95% prediction limits are based on Wald statistical tests. We used SAS software version 9.4 (SAS, Inc., Cary, NC) to apply data entry, processing, and statistical analysis. Statistical significance was achieved when *P* was <.05.

## 3 Results

Patients with dengue may arrive at the hospital shortly to obtain optimal triage and management of the ED. The LOS of ED triage patients is defined as the time of arrival to time of discharge as documented in the electronic medical records. Table 1 shows a total of 92,314, 100,735, and 100,214 for the ED visits from this tertiary hospital, and the LOS among ED triaged patients from these three major divisions in 2014, 2015, and 2016. Over the 3 years, the majority of ED visits occurring in this tertiary hospital were 47.4%, 48.6%, and 48.0% of those admitted to the division of medicine. The RCS was initiated in August 2015, when the dengue outbreak started and the outbreak was severe. The ED admission visits (13,714) of the division of medicine in the 2015 outbreak were apparently higher than those (10,861) of the previous dengue season in 2014. The average LOS of the division of medicine during the dengue-outbreak season (581.5 minutes) was significantly higher than the LOS in the non-dengue (531.3 minutes) season; however, in the previous year, the average LOS of the division of medicine during the dengue (602.8 minutes) or non-dengue (591.2 minutes) season was higher than the LOS in the outbreak year. Additionally, the average LOS for the overall ED population was 439.0 during the dengue season, significantly higher than the 425.8 minutes observed during the non-dengue season. The LOS for both dengue and non-dengue months in 2014 was higher than for the LOS in 2015 when there was an outbreak. The average LOS for the dengue-outbreak and non-dengue seasons was 411.6 and 384.9 minutes, respectively. A prolonged LOS for ED patients was observed during the dengue season in 2014 and the 2015 outbreak. After the RCS was set up in 2015, the LOS during the dengue season was 308.8, much lower than the 332.2 that was during the non-dengue season in 2016.

**Table 1:** Length of stay (minutes) in the ED for the 3 major divisions and seasons of a tertiary hospital from 2014 to 2016.

Year	Division	N	Mean	SD	95% CI for mean		*P* ^∗^
2014	Medicine (nontrauma, adult)						
	Dengue	10,861	602.8	982.2	584.4	621.3	.263
	Non-dengue	32,940	591.2	797.6	582.6	599.8	
	Trauma, adult						
	Dengue	5882	320.6	514.2	307.4	333.7	.208
	Non-dengue	17,577	309.0	836.2	296.6	321.4	
	Pediatrics, nontrauma						
	Dengue	3104	242.7	389.6	229	256.4	.152
	Non-dengue	10,952	231.2	407.1	223.6	238.8	
	Others^†^						
	Dengue	2778	268.2	579.0	246.7	289.8	.763
	Non-dengue	8220	272.1	600.0	259.1	285.1	
	Overall						
	Dengue	22,625	439.0	786.9	428.7	449.2	.027
	Non-dengue	69,689	425.8	755.4	420.2	431.4	
	Total	92,314	429.0	763.3	424.1	434.0	
2015	Medicine (nontrauma, adult)						
	Dengue-outbreak	13,714	581.5	799.3	568.1	594.9	<.0001
	Non-dengue	35,291	531.3	701.7	524.0	538.7	
	Trauma, adult						
	Dengue-outbreak	5926	309.9	513.4	296.8	322.9	<.0001
	Non-dengue	17,666	278.7	459.1	272.0	285.5	
	Pediatrics, nontrauma						
	Dengue-outbreak	4304	204.9	325.1	195.2	214.6	.080
	Non-dengue	10,987	215.4	351.3	208.8	222.0	
	Others^†^						
	Dengue-outbreak	4089	206.8	459.2	192.7	220.9	.101
	Non-dengue	8758	221.3	484.4	211.2	231.5	
	Overall						
	Dengue-outbreak	28,033	411.6	666.5	403.8	419.4	<.0001
	Non-dengue	72,702	384.9	598.2	380.5	389.2	
	Total	100,735	392.3	618.1	388.5	396.1	
2016	Medicine (nontrauma, adult)						
	Dengue	11,782	424.3	553.5	414.3	434.3	<.0001
	Non-dengue	36,312	463.3	631.2	456.8	469.7	
	Trauma, adult						
	Dengue	6131	225.3	374.7	215.9	234.7	.0007
	Non-dengue	17,729	244.4	397.7	238.6	250.3	
	Pediatrics, nontrauma						
	Dengue	3561	140.9	188.2	134.7	147.1	<.0001
	Non-dengue	12,311	168.9	231.5	164.8	173	
	Others^†^						
	Dengue	2891	221.8	469.2	238.9	469.2	.121
	Non-dengue	9497	206.3	472.4	215.8	472.4	
	Overall						
	Dengue	4365	308.8	477.5	302.8	314.8	<.0001
	Non-dengue	75,849	332.2	529.8	328.4	335.9	
	Total	100,214	326.5	517.6	323.3	329.7	

CI = confidence interval, ED = emergency department, LOS = length of stay, N = number, SD = standard deviation.

∗ Student *t* test.

† Admissions to the ED due to other reasons such as dermatologic, gynecology/obstetrics, and so on.

The logistic regression analysis revealed that the shorter LOS in the ED has a significantly higher performance of case-treatment rates compared to longer LOS (>24 hours) in Table 2. Odds ratios (ORs) with 95% confidence intervals (CIs) for LOS ≤2 hours, 2 < LOS ≤ 6 hours, 6 < LOS ≤ 24 hours, and LOS >24 hours were 12.60 (95% CI: 12.38–12.83), 7.74 (95% CI: 7.60–7.88), and 4.44 (95% CI: 4.35–4.52), respectively. Although the dengue outbreak resulted in a lack of mobilization of personnel and spare space in the ED, there was no statistical difference in case-treatment rates between dengue season (OR = 1.00, 95% CI: 0.99–1.01) and dengue-outbreak season (OR = 1.00, 95% CI: 0.9–1.02) compared to non-dengue season. Therefore, a proper response to RCS settings from the hospital administration would ameliorate the work overload of the staff and decrease the crowding of the ED patients.

**Table 2:** Effect estimates of case-treatment rates of ED patients with LOS during 2014 January and 2016 September.

Effect	OR (95% CI^∗^)	*P* ^†^
ED LOS, h		
≤2	12.601 (12.375–12.832)	<.0001
>2 and ≤6	7.738 (7.597–7.881)	<.0001
>6 and ≤24	4.435 (4.352–4.520)	<.0001
>24	1.000	
Season		
Dengue^‡^	1.000 (0.987–1.014)	.973
Dengue-outbreak^§^	0.999 (0.985–1.015)	.943
Non-Dengue	1.000	

CI =confidence interval, ED = emergency department, LOS = length of stay, OR = odds ratio.

∗ Wald confidence limits.

† Analysis of maximum likelihood estimates.

‡ August to October, 2014 and 2016.

§ August to October, 2015.

**Figure 2:**
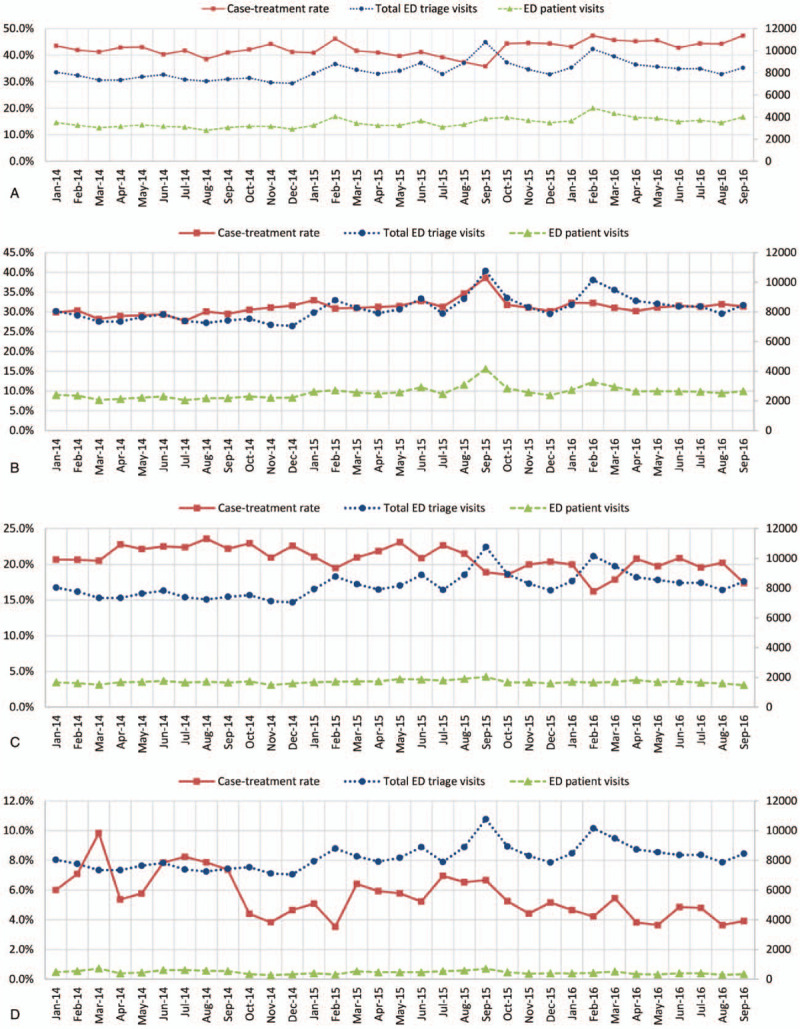
Monthly case-treatment rates (orange line with squares, left axis) of registered ED patient visits, and the total numbers of registered and triaged ED patient visits for 4 categories of LOS, shown in blue and gray lines (right axis), during Dengue outbreak (red grid) and 2014–2016 September. (A) LOS ≤ 2 hours. (B) 2 < LOS ≤ 6 hours. (C) 6 < LOS ≤ 24 hours. (D) LOS > 24 hours. ED = emergency department, LOS = length of stay.

Figure 2 illustrates the diagrams for ED patient visits with respect to 4 categories of the LOS, that is, ≤2 hours, 2 < LOS ≤ 6 hours, 6 < LOS ≤ 24 hours, and >24 hours, respectively. The registered and triaged ED patient visits (gray lines) and the total number of registered and triaged ED patient visits (gray lines) were markedly increased during a dengue outbreak (red grid) in 2015. In particular, the monthly case-treatment rates were affected and decreased for LOS ≤2 hours (Fig. 2A).

## 4 Discussion

The study proposes an independent unit to initiate an RCS that can be used to improve the triage process in the overcrowding of the EDs. With the assistance of administrative and clinical protocols, it is the first study to investigate the effects of a RCS on objective measures of LOS during a severe dengue outbreak. Setting up the RCS outside the regular emergency department during a dengue epidemic would decrease the delay of the large patient census flow, mitigate the possible stress of the health care professionals, and improve the efficiency of patient management in the ED.

Although the clinical care no longer relies on the performance of individual physicians, patients need a diverse medical team of professionals working together in order to achieve the quality requirements of modern medicine. The use of quality control tools was to achieve the quality of medical care during the sophisticated integral approach.^[14]^ From the patient's perspective, the total time spent waiting for the test results and being seen by a physician may create a feeling of the blocking and overcrowding of the ED. The core mission of the ED was to care for acute illness and injury patients in a short time; EPs were committed to providing high-quality emergency care as quickly as possible to all patients; but the sharp rise in the number of emergency patients during dengue outbreaks limited everyone's access to timely emergency care and resulted in the ED crowding and boarding when insufficient hospital capacity is available for admitted patients to be transferred to inpatient beds, and jeopardized emergency medical care and patient safety.^[15–18]^

Fast track ED services have been proposed and implemented in many countries, such as the United States^[18]^ and Australia,^[19]^ to ensure timely care and improve the ED crowding and shorten the LOS. Our study results support the implementation that fast track ED services^[19]^ which are used in health care in emergency medicine for minor illnesses and injuries can shorten the LOS of ED patients and reduce the likelihood of relapse. Usually, low-complexity patients do not use the same treatment spaces as sicker patients, and the resources they need are generally simple and readily available.^[20]^ The study adopted similar concepts by using non-EPs and nurses from other clinical departments of the hospitals with standard treatment protocols to relieve the noncritical high census of patient flow during the dengue outbreak and maintain ED performance during dengue outbreaks. Although the patient census increased to nearly twice the regular time (Fig. 2), the ED was still able to handle the critical patients and maintain the LOS at earlier levels.

Effective administrative preparedness and response are regarded as the most important issues for certain public health events, and hospitals are required to have preparedness and response plans for these events. Comparing this to an infectious disease outbreak, which might have a high patient census for a certain time, the mass casualty incident (MCI) lasted for a short period, and the ED usually could properly respond to these events by initiating the management protocol of MCI. If these events were to occur continuously, that is, influenza or dengue outbreak, longer optimal administrative plans are suggested to be carried out to support the critical function of the healthcare facilities, especially for the ED. Our study results proposed that the ED performance during the dengue season did not definitely worse than the performance during the non-dengue seasons, and that there was a significant improvement in preparation for the RCS. We also indicated that mean LOS in the hospital did not prolong over the course of time. Few more clinical loading shifts incurred by the overcrowding in ED occurred during the dengue outbreak, and most of the minor dengue patients were quickly treated in the RCS. The previous study in the 2009 novel influenza season also supported setting up rapid flu clinic service that was beneficial to health care facilities during certain infectious disease outbreak seasons.^[10]^ Therefore, every tertiary-care center should be encouraged to monitor the patient census and LOS in the ED to prepare for emergencies of MCI or infectious disease, not only by pharmacists stockpiling and creating space for certain disease wards, but also formulating and supporting infection control task forces, specifically designed to oversee and manage the workloads of health care workers and manage hospital responses.^[21]^

Implementing the rapid clinic services in time during a disease outbreak may be difficult. The Daily Emergency Department Surveillance System has suggested that the ED based surveillance systems have great impacts on public health because the utilization of the ED in certain events, such as mass gatherings, injuries, terrorism, and so on, is not limited to the ED itself. ED based surveillance is regarded as one of the most important public health surveillance systems for bioterrorism and infectious diseases.^[22]^ The ED-based surveillance system adapted *International Classification of Diseases, 9th revision, clinical modification* (*ICD-9 CM* code) for ED visits into 11 different syndromic groups important in Taiwan for a long time.^[23]^ Although the system has identified several influenza and enterovirus outbreaks in the past year; the evidence on relieving the ED overcrowding or early warning to the first line EDs was limited. Our research hospital had launched the RCS within 2 weeks after the large scale of the outbreak and had proven an improvement in case-treatment and shortened the LOS for minor illness patients, those usually staying in the ED for <2 hours; however, the case-treatment rate still remained lower for patients with severe or moderate or mild conditions who needed further management and disposition. The heat wave experienced in France also suggested impacts on response and preparedness of health care facilities, indicating that previous experiences with public health surveillance would have additional backup staff and available wards, and represented the importance of real time public health surveillance.^[24]^ This is crucial for hospital preparedness, the planning, and training of the RCS, and for infectious disease outbreaks and other emergencies. Therefore, further analyses of timing for initiating the backup plans and proposing routine preparedness and responses for tertiary hospitals should be carried out to manage and qualify the workload of the health care workers and improve patient safety in the ED.^[25]^

One of the limitations of the present study is that the workload management of the health care workers and the improvement of patient safety in the ED were not quantified in our analyses.

## 5 Conclusions

In this study, an early intervention led to a rapid amelioration of dengue case management and maintenance of case-treatment performance during an endemic dengue period. Further planning and training of the RCS was essential to health care facilities and could be crucial for hospital preparedness for infectious disease outbreaks. LOS could be an indicator that novel services are being evaluated in the ED. Routine monitoring and daily practice should be considered to initiate rapid clinical treatment to relieve ED crowding and human resource deployment.

## Author contributions

**Administrative support:** Chih-Chia Hsieh.

**Collection and assembly of data:** Hsin-I Shih, Yi-Ting Huang, and Tzu-Ching Sung.

**Conception and design:** Hsin-I Shih and Tzu-Ching Sung.

**Conceptualization:** Tzu Ching Sung.

**Data analysis and interpretation:** Hsin-I Shih and Tzu-Ching Sung.

**Final approval of manuscript:** All authors.

**Formal analysis:** Tzu Ching Sung.

**Manuscript writing:** Hsin-I Shih and Tzu-Ching Sung.

**Writing – original draft:** Tzu Ching Sung.

## References

[1] Halstead SB. Dengue. Lancet 2007;370:1644–52. 17993365 10.1016/S0140-6736(07)61687-0

[2] WHO. Dengue: Guidelines for Diagnosis, Treatment, Prevention and Control: New Edition. WHO Guidelines Approved by the Guidelines Review Committee. Geneva: World Health Organization Press; 2009. 23762963

[3] WHO. Handbook for Clinical Management of Dengue. Geneva: World Health Organization Press; 2012.

[4] Huy NT Van Giang T Thuy DH. Factors associated with dengue shock syndrome: a systematic review and meta-analysis. PLoS Negl Trop Dis 2013;7:e2412. 24086778 10.1371/journal.pntd.0002412 PMC3784477

[5] Disease Control Division DoH, Kaohsiung City Government Dengue Fever Control in Kaohsiung City. In:2015.

[6] Center for Disease Control T. Taiwan National Infectious Disease Statistics System. Available at: http://nidss.cdc.gov.tw/en/?treeid = 00ed75d6c887bb27&nowtreeid = d39475c2db7cd87b. Published 2016. Accessed 10/Dec/2016, 2016.

[7] Chan L Reilly KM Salluzzo RF. Variables that affect patient throughput times in an academic emergency department. Am J Med Qual 1997;12:183–6. 9385728 10.1177/0885713X9701200403

[8] Rathlev NK Obendorfer D White LF. Time series analysis of emergency department length of stay per 8-hour shift. West J Emerg Med 2012;13:163–8. 22900106 10.5811/westjem.2011.7.6743 PMC3415804

[9] Chen LS Wang YR. A conceptual framework for Taiwan's hospital clinical performance indicators. J Formos Med Assoc 2015;114:381–3. 25749109 10.1016/j.jfma.2015.01.021

[10] Shih HI Ho TS Chang CM. Impacts of rapid flu clinic services at an emergency department during the pandemic flu season. Am J Infect Control 2012;40:165–9. 21775019 10.1016/j.ajic.2011.03.006 PMC7115284

[11] Sung SF Huang YC Ong CT. Validity of a computerised five-level emergency triage system for patients with acute ischaemic stroke. Emerg Med J 2013;30:454–8. 22736717 10.1136/emermed-2012-201423

[12] Diagnostics S. SD BIOLINE Dengue Duo In: Diagnostics S, ed. Kyonggi-do, Korea 2016.

[13] Shih HI Hsu HC Wu CJ. Applications of a rapid and sensitive dengue DUO rapid immunochromatographic test kit as a diagnostic strategy during a Dengue type 2 epidemic in an urban city. PLoS One 2016;11:e0158437. 27415767 10.1371/journal.pone.0158437 PMC4945082

[14] Horwitz LI Green J Bradley EH. US emergency department performance on wait time and length of visit. Ann Emerg Med 2010;55:133–41. 19796844 10.1016/j.annemergmed.2009.07.023 PMC2830619

[15] Bernstein SL Verghese V Leung W. Development and validation of a new index to measure emergency department crowding. Acad Emerg Med 2003;10:938–42. 12957975 10.1111/j.1553-2712.2003.tb00647.x

[16] Pines JM Prabhu A Hilton JA. The effect of emergency department crowding on length of stay and medication treatment times in discharged patients with acute asthma. Acad Emerg Med 2010;17:834–9. 20670320 10.1111/j.1553-2712.2010.00780.x

[17] McCarthy ML Zeger SL Ding R. Crowding delays treatment and lengthens emergency department length of stay, even among high-acuity patients. Ann Emerg Med 2009;54:492–503. e4. 19423188 10.1016/j.annemergmed.2009.03.006

[18] Pines JM Hilton JA Weber EJ. International perspectives on emergency department crowding. Acad Emerg Med 2011;18:1358–70. 22168200 10.1111/j.1553-2712.2011.01235.x

[19] Considine J Kropman M Kelly E. Effect of emergency department fast track on emergency department length of stay: a case-control study. Emerg Med J 2008;25:815–9. 19033498 10.1136/emj.2008.057919

[20] Schull MJ Kiss A Szalai JP. The effect of low-complexity patients on emergency department waiting times. Ann Emerg Med 2007;49:257–64. 17049408 10.1016/j.annemergmed.2006.06.027

[21] Shakoor S Mir F Zaidi AK. Hospital preparedness in community measles outbreaks-challenges and recommendations for low-resource settings. Emerg Health Threats J 2015;8:24173. 25882388 10.3402/ehtj.v8.24173 PMC4400300

[22] Paladini M. Daily emergency department surveillance system—Bergen County, New Jersey. MMWR Suppl 2004;53:47–9. 15714627

[23] Wu TS Shih FY Yen MY. Establishing a nationwide emergency department-based syndromic surveillance system for better public health responses in Taiwan. BMC Public Health 2008;8:18. 18201388 10.1186/1471-2458-8-18 PMC2249581

[24] Crabbe C. France caught cold by heatwave. Bull World Health Organ 2003;81:773–4. 14758441 PMC2572322

[25] Singer AJ Thode HC Jr Viccellio P. The association between length of emergency department boarding and mortality. Acad Emerg Med 2011;18:1324–9. 22168198 10.1111/j.1553-2712.2011.01236.x

